# Assessment of spontaneous breathing during pressure controlled ventilation with superimposed spontaneous breathing using respiratory flow signal analysis

**DOI:** 10.1007/s10877-020-00545-4

**Published:** 2020-06-13

**Authors:** Stefan Kreyer, William L. Baker, Vittorio Scaravilli, Katharina Linden, Slava M. Belenkiy, Corina Necsoiu, Thomas Muders, Christian Putensen, Kevin K. Chung, Leopoldo C. Cancio, Andriy I. Batchinsky

**Affiliations:** 1 Department of Anesthesiology and Intensive Care Medicine, University Hospital Bonn, Bonn, Germany; 2 U.S. Army Institute of Surgical Research, JBSA Fort Sam Houston, San Antonio, TX USA; 3 Department of Anesthesia, Critical Care and Emergency, Fondazione IRCCS Ca’ Granda - Ospedale Maggiore Policlinico, Milano, MI Italy; 4 Pediatric Department, University Hospital Bonn, Bonn, Germany; 5 Department of Anesthesiology, West Virginia University School of Medicine, Morgantown, WV USA; 6 Department of Medicine, Uniformed Services University, Bethesda, MD USA; 7 The Geneva Foundation, Tacoma, WA USA

**Keywords:** APRV, BIPAP, Spontaneous breathing, ARDS, Breath separation

## Abstract

Integrating spontaneous breathing into mechanical ventilation (MV) can speed up liberation from it and reduce its invasiveness. On the other hand, inadequate and asynchronous spontaneous breathing has the potential to aggravate lung injury. During use of airway-pressure-release-ventilation (APRV), the assisted breaths are difficult to measure. We developed an algorithm to differentiate the breaths in a setting of lung injury in spontaneously breathing ewes. We hypothesized that differentiation of breaths into spontaneous, mechanical and assisted is feasible using a specially developed for this purpose algorithm. Ventilation parameters were recorded by software that integrated ventilator output variables. The flow signal, measured by the EVITA® XL (Lübeck, Germany), was measured every 2 ms by a custom Java-based computerized algorithm (Breath-Sep). By integrating the flow signal, tidal volume (V_T_) of each breath was calculated. By using the flow curve the algorithm separated the different breaths and numbered them for each time point. Breaths were separated into mechanical, assisted and spontaneous. Bland Altman analysis was used to compare parameters. Comparing the values calculated by Breath-Sep with the data from the EVITA® using Bland–Altman analyses showed a mean bias of − 2.85% and 95% limits of agreement from − 25.76 to 20.06% for MV_total_. For respiratory rate (RR) RR_set_ a bias of 0.84% with a SD of 1.21% and 95% limits of agreement from − 1.53 to 3.21% were found. In the cluster analysis of the 25th highest breaths of each group RR_total_ was higher using the EVITA®. In the mechanical subgroup the values for RR_spont_ and MV_spont_ the EVITA® showed higher values compared to Breath-Sep. We developed a computerized method for respiratory flow-curve based differentiation of breathing cycle components during mechanical ventilation with superimposed spontaneous breathing. Further studies in humans and optimizing of this technique is necessary to allow for real-time use at the bedside.

**Electronic supplementary material:**

The online version of this article (10.1007/s10877-020-00545-4) contains supplementary material, which is available to authorized users.

## Introduction

Mechanical ventilation (MV) is used in the treatment of acute respiratory distress syndrome (ARDS) to secure ventilation and oxygenation [1] and is based on positive-pressure delivery of air into the lungs. Although life sustaining, mechanical ventilation can also cause damage in the form of ventilator-induced lung injury (VILI) [2]. In contrast, low-tidal-volume ventilation has led to higher survival rates [3–5], especially with implementation of reduced driving pressure [6, 7]. Often, increased sedatives or even paralytics are required to optimize synchrony between the patient and the ventilator. This eliminates the natural ability to adjust to metabolic requirements, deconditions the diaphragm and respiratory musculature [8].

One method for improving synchrony and reducing the invasiveness of mechanical ventilation, spontaneous breathing (SB), can be integrated into ventilation patterns, e.g. by using Biphasic Positive Airway Pressure (BIPAP) or Airway Pressure Release Ventilation (APRV). There are several important differences between positive-pressure induced mechanical ventilation and unsupported spontaneous breathing [9, 10]. Spontaneous ventilation involves use of the diaphragm under negative pressure and, as such, is more evenly distributed to the dependent regions of the lung [11, 12]; spontaneous breathing is usually more variable in frequency and depth as it matches the metabolic requirements of the moment. Importantly, spontaneous breathing occurs with little or no sedation and permits natural airway mucus clearance, conditions the diaphragm and enables early ambulation and discharge from the intensive care unit (ICU). Diaphragm atrophy is associated with prolonged ventilation, prolonged ICU admission and higher risk of complications [13].

**Fig. 1:**
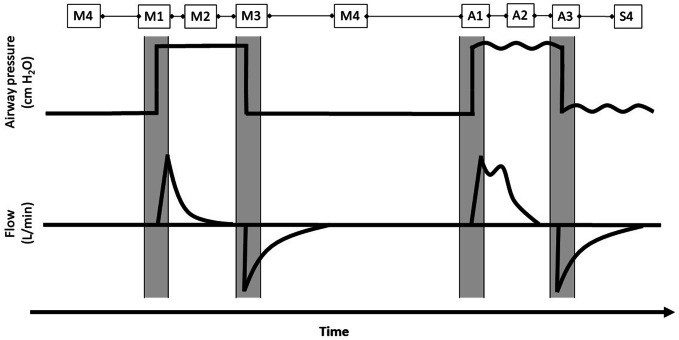
Two schematic breathing cycles in BIPAP/APRV. 1: Change from lower PEEP-level (P_low_) to upper PEEP-level (P_high_). 2: Upper PEEP-level (P_high_). 3: Change from lower PEEP-level (P_low_) to upper PEEP-level (P_high_). 4: Lower PEEP-level (P_low_). M1–M4: No spontaneous breathing is present: mechanical ventilation (pressure controlled). A1–A3: Spontaneous breathing is present: assisted breathing. S4: Spontaneous breathing is present: spontaneous breathing

BIPAP/APRV is a mode of pressure-controlled ventilation which switches between two levels of positive pressure (Fig. 1), thereby generating a pressure difference resulting in air flow. This allows the patient to breath spontaneously in every phase of the ventilation cycle. Integrating SB leads to higher cardiac output and oxygen delivery (DO_2_) [8], higher regional perfusion [14], reduced need for inotropes and vasopressors, [8] and improved ventilation-perfusion matching [11]. On the other hand, inadequate SB has the potential to worsen lung injury [15, 16].

Exact measurement of SB during BIPAP/APRV can be complex but is important due to the potential deleterious side effects of inadequate SB. Currently available mechanical ventilators classify each breath as either mechanical or spontaneous, ignoring the reality that such classification of a breath is idealized and often highly imprecise. The assisted breaths (neither spontaneous nor mechanical) are especially difficult to measure and are not displayed as an independent entity in ventilators [17]. However, these assisted breaths may be a major part of the breathing cycle and neglecting them may result in inaccurate assessment of respiratory values. In order to solve the problem of SB differentiation, we designed an algorithm to differentiate the parts of the breathing cycle and used it on data we previously collected in an animal model of smoke and burn injury comparing an extracorporeal carbon dioxide removal (ECCO_2_R) device with standard ventilation [18]. We hypothesized that measurement and differentiation of the type of spontaneous breath using ARPV/BIPAP is feasible by implementing our algorithm.

## Methods and materials

This study was approved by the U.S. Army Institute of Surgical Research Institutional Animal Care and Use Committee (Protocol A-13-012). It was conducted in compliance with the Animal Welfare Act and the implementing Animal Welfare Regulations and in accordance with the principles of the *Guide for the Care and Use of Laboratory Animals*.

A detailed description of methods was previously published [18]. Briefly, 15 non-pregnant farm-bred ewes were used for this study. After induction of smoke inhalation injury and thermal burns [19] under complete surgical plane anesthesia, sheep were monitored in the animal ICU around-the-clock and allowed to wake up. Next, analgesia was provided by a continuous infusion of fentanyl and midazolam titrated to effect. The sheep were allowed to stand, drink water and eat hay/pellets ad libitum.

The animals were ventilated with the Dräger Evita® XL ventilator (Dräger Medical, Lübeck, Germany) in volume-control mode with a tidal volume (V_T_) of 15 mL/kg until onset of ARDS.

After the onset of ARDS, defined as PaO_2_/F_i_O_2_-ratio (PFR) < 300 over one hour [20], ventilator settings were switched to APRV-mode with BIPAP settings [21], without pressure support. Time_high_:Time_low_ was 1:2. P_high_ was set to achieve a V_T_ 6–8 mL/kg. Positive end expiratory pressure (PEEP) (P_low_) and fraction of inspired oxygen (F_i_O_2_) were titrated based on the ARDSNet mechanical ventilation protocol (lower PEEP/higher F_i_O_2_ settings) [5, 22]. The respiratory rate (RR) was set to achieve a PaCO_2_ < 55 mmHg. If PaCO_2_ reached a level above 55 mmHg and/or PaO_2_ was below 70 mmHg despite a RR = 35/min, the P_high_ was increased stepwise, resulting in higher V_T_. The V_T_ was checked every 10 min or more frequently, and if necessary P_high_ was adjusted to achieve the goal V_T_.

After the onset of ARDS, animals were randomized into two groups: Control (C) (n = 8) and extracorporeal carbon dioxide removal (ECCO_2_R) carried out with the Hemolung (Alung Technologies, Pittsburgh, PA) (n = 7). For a detailed description we refer to our previously published manuscript [18].

In addition to the waveform data recorded by the Integrated Data Exchange and Archival (IDEA), hourly values for ventilator settings were recorded by the care provider. Ventilator data recorded every 3 h were used for analysis. The reference point for this was the time of ARDS onset.

If animals survived to the end of study, 72 h after injury induction, they were euthanized in accordance with the American Veterinary Medical Association’s *Guidelines for the Euthanasia of Animals*, 2013.

### Ventilation analysis

**Fig. 2:**
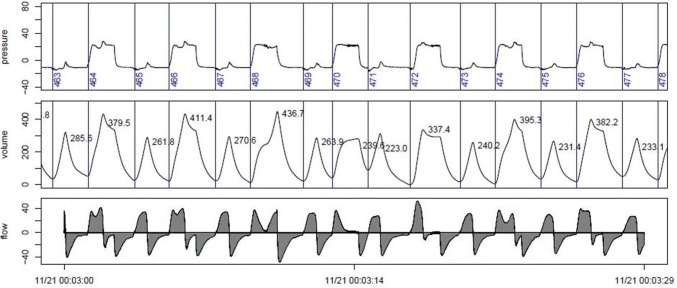
Exemplary JPEG graphical file displaying the airway pressure (cm H_2_O), derived volume curve (mL) and measured flow curve (L/min)

During the experiment, ventilation parameters were recorded by means of custom software (IDEA) and stored for offline analysis. The flow measured by the EVITA® XL was used for ventilation analysis. The flow signal was measured every 2 ms. by a custom Java-based computerized algorithm. By integrating the flow signal, the V_T_ of each breath was calculated. A JPEG graphical file displaying the airway pressure (P_AW_) derived volume curve and measured flow curve was generated (Fig. 2). By using the flow curve, the algorithm separated the different breaths and numbered them for each time point. For a detailed description of the algorithm we refer to the supplemental digital content, that describes the automatic algorithm. For each timepoint, a 20-min interval of data was extracted consisting of 10 min before and after the timepoint. From the 20-min of extracted data, three minutes of clean data closest to the timepoint of arterial blood gas (ABG) analysis was selected and analyzed. If at this time the signal was not clear or a separation of different breaths was not possible, the next closest three-minute interval was taken. If we could not find clear three-minute interval within 10 min before the timepoint, the timepoint was excluded in this analysis. Data were manually analyzed and compared by two researchers. The derived breaths were grouped into the following categories: mechanical, spontaneous and assisted breaths. Spontaneous breathing on P_low_ was defined as spontaneous breathing. Spontaneous breathing on P_high_, on the change of P_low_ to P_high_ and on the change of P_high_ to P_low_ was defined as assisted breathing. If no spontaneous breathing occurred on P_high_ it was defined as mechanical breathing (see Fig. 1 for explanation). The mean for all three types of breaths during the three-minute period was calculated. Respiratory rate was separated into mechanical (RR_mech_), assisted (RR_assist_) and spontaneous (RR_spont_). RR_set_ was the sum of mechanical and assisted breaths. RR_spont_ included only spontaneous breaths. Minute volume (MV) was calculated as RR multiplied by V_T_ for the whole 3 min divided by 3. Sometimes the animals were breathing asynchronous or with a high respiratory rate, resulting in a situation that a spontaneous breath followed a mechanical breath or vice versa in a close distance, meaning the breaths were not independent. The algorithm did count every breath as own entity (spontaneous and mechanical), although in reality it was an assisted breath. As a result, the tidal volume of the subsequent breath was measured incorrectly. As the algorithm was unable to detect this situation, we had to correct/reclassify these breaths manually as assisted breath and merged the two breaths.

Several factors influenced the decision to merge breaths. A simple time cut off was not enough to make this decision, because at a higher RR these numbers would have been much lower. If the previous breath influenced the following breath they were merged, meaning if the pressure/flow curve showed the influence of the previous breath on the subsequent breath we merged them. In our data analysis we used the inspiratory flow to measure V_T_ and for the classification of the breaths. As a last step we used the formula (V_T insp_ − V_T exp_)/ V_T insp_ * 100. High values compared to the rest of the same 3 min step gave a sign to merge two breaths. This procedure was used as a verification step for the definition of the breath.

### Statistics

JMP®14.0.0, SAS Institute Inc. was used for statistical analysis and GraphPadPrism® Version 5.02, GraphPad Software, Inc., USA was used for Bland-Altmann analysis and graph preparation. Data were checked for normal distribution using Shapiro-Wilks test. Non-normal distributed data were analyzed using a Kruskal–Wallis test with Dunn’s multiple comparison test. For comparison of the ventilation data from the two methods (EVITA® versus our algorithm) delivered values for respiratory rate total, respiratory rate spontaneous, and minute ventilation were compared using a Bland–Altman analyses.

To evaluate the effect of different proportions of spontaneous breathing on the comparison of both methods a cluster analysis was performed. Therefore, the 153 recorded timepoints were ranked according to their proportion of mechanical, spontaneous, and assisted breathes, respectively. Method-comparison was then done separately for each cluster using the 25 timepoints (16,3%) with the highest proportion of each type of ventilation (mechanical, spontaneous, and assisted), respectively.

Cluster analyses was necessary for individual animals, due to the physiology of ARDS development over time and the amount of spontaneous breathing being different at each timepoint/animal. The cluster analysis separated the amount of spontaneous breathing with lowest amount in the mechanical cluster, followed by the assisted cluster and with highest amount of spontaneous breathing in the spontaneous cluster.

## Results

Twenty animals and 931 h of ICU time were required to complete this study. Five animals were excluded due to either insufficient lung injury (n = 3) or severe/nonsurvivable ARDS (n = 2). A total of 153 timepoints was available for analysis.

Comparing the values of Breath-Sep with the data from the EVITA® for MV_total_ using Bland–Altman analyses we found a bias of − 2.85% with 95% limits of agreement from − 25.76 to 20.06%.

**Fig. 3:**
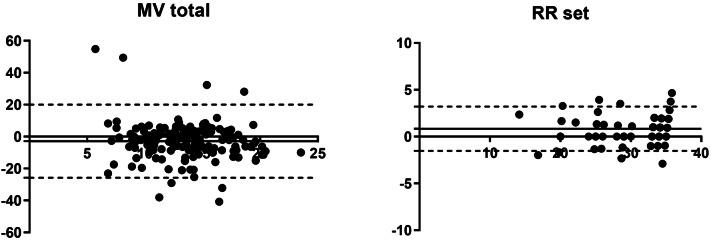
Bland Altman analysis. Bland–Altman plot for all timepoints of percentage of minute ventilation and respiratory rate set measured with the EVITA and offline flow analysis. Solid line is bias and dashed lines are 95% limits of agreement. x-axis: L/min (MVtotal) and respiratory rate, y-axis: percentage of difference

Comparing the values of Breath-Sep with the data from the EVITA® for RR_set_ we found a bias of 0.84% and 95% limits of agreement from − 1.53 to 3.21% (Fig. 3).

Assisted breaths, as defined in our methods, was observed in 45 ± 19.2% of the breathing cycle (mean ± SD), while mechanical breaths were observed in 24.6% ± 25.6% and spontaneous breaths in 30.4% ± 16.5%.

Values of V_T_/kg and percentage of assisted, mechanical and spontaneous breaths for all animals and timepoints are presented as median and 25/75 percentile (Table 1).

**Table 1:** Values for assisted, mechanical and spontaneous breaths for all animals and timepoints

	Assisted (a)	Mechanical (m)	Spontaneous (s)	P	a _vs._ m	a _vs._ s	m _vs._ s
V_T_/kg (mL/kg)	10.4 (8.9;11.6)	6.8 (6;8.3)	6.5 (5;8.5)	< 0.0001	< 0.05	< 0.05	ns
Percentage (%)	46.5 (32.7;57.4)	14.6 (6.3;33.2)	36 (21.6;41)	< 0.0001	< 0.05	< 0.05	< 0.05

Values are presented as median and (25;75) percentile

**Fig. 4:**
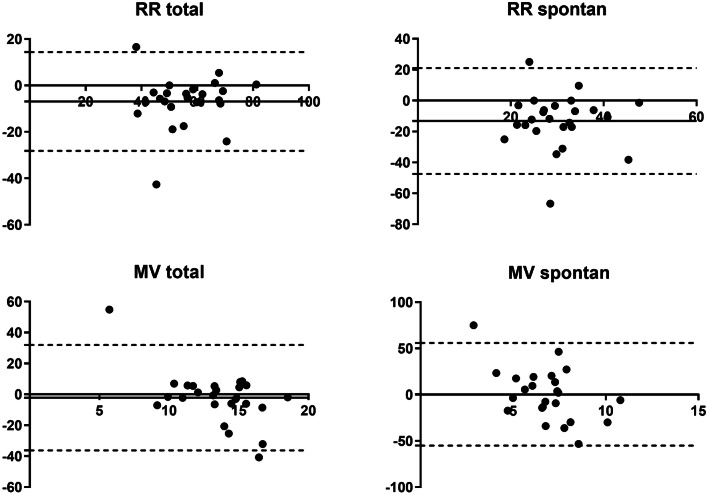
Bland–Altman plot for the 25 highest timepoints of percentage of spontaneous ventilation. Bland–Altman plot for the 25 highest timepoints of percentage of spontaneous ventilation. Analysis of respiratory rate total, respiratory rate spontaneous, minute ventilation total and minute ventilation spontaneous measured with the EVITA and offline flow analysis. Solid line is bias and dashed lines are 95% limits of agreement. x-axis: L/min (MVtotal and MVspontan) and respiratory rate, y-axis: percentage of difference

**Fig. 5:**
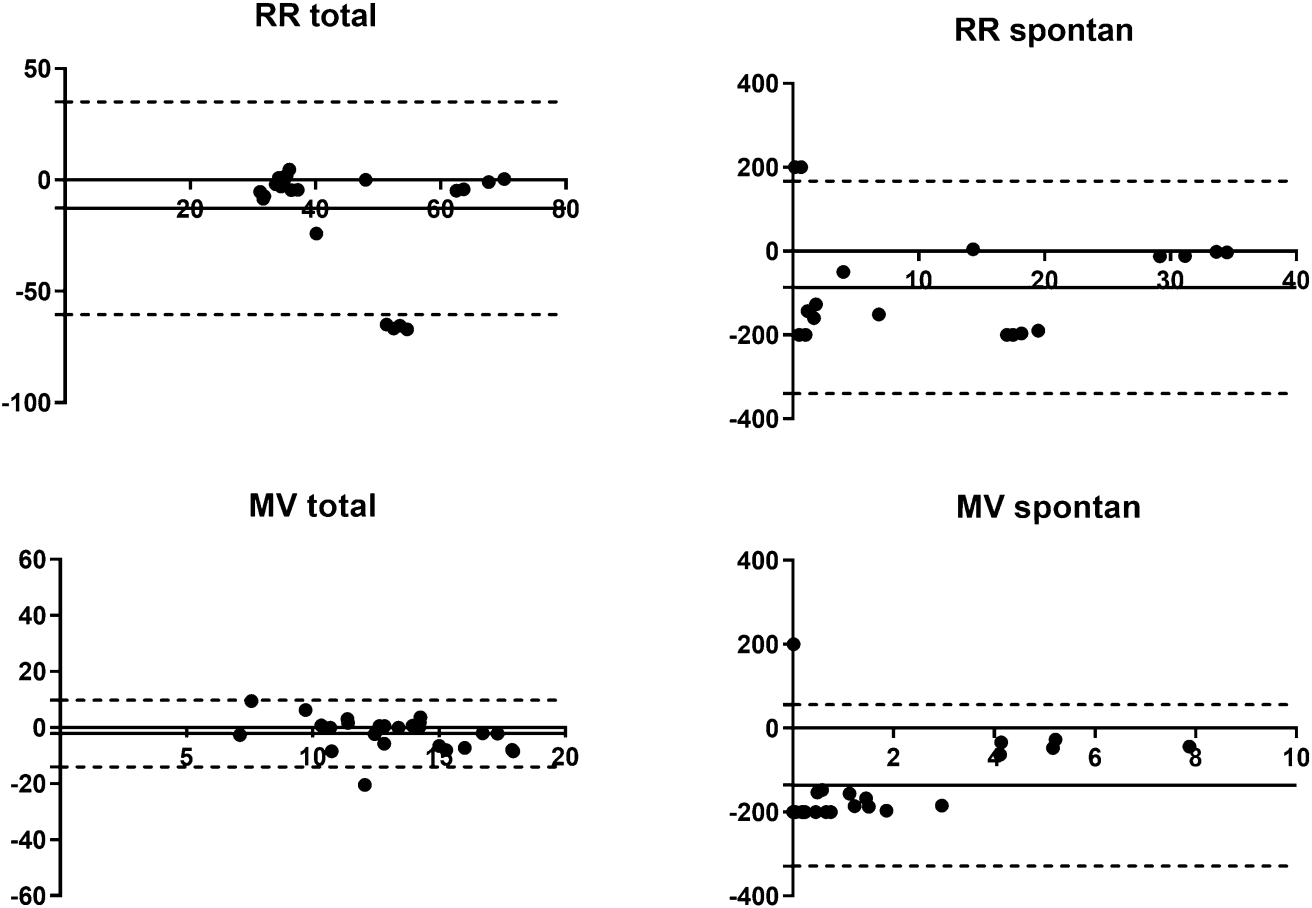
Bland–Altman plot for the 25 highest timepoints of percentage of mechanical ventilation. Bland–Altman plot for the 25 highest timepoints of percentage of mechanical ventilation. Analysis of respiratory rate total, respiratory rate spontaneous, minute ventilation total and minute ventilation spontaneous measured with the EVITA and offline flow analysis. Solid line is bias and dashed lines are 95% limits of agreement. x-axis: L/min (MVtotal and MVspontan) and respiratory rate, y-axis: percentage of difference

**Fig. 6:**
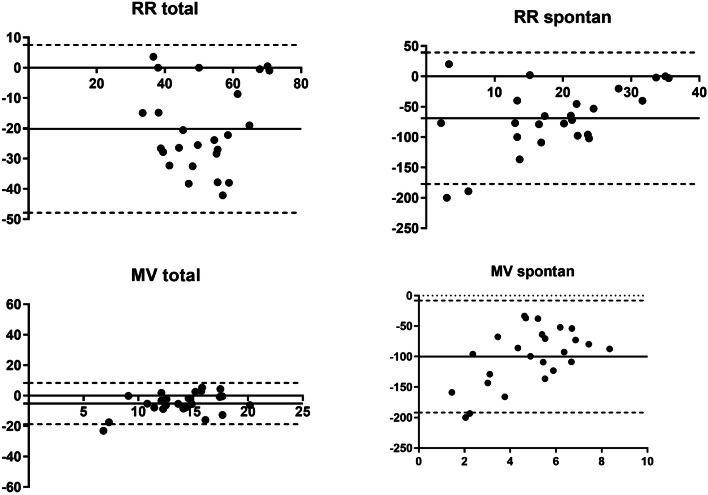
Bland–Altman plot for the 25 highest timepoints of percentage of assisted ventilation. Bland–Altman plot for the 25 highest timepoints of percentage of assisted ventilation. Analysis of respiratory rate total, respiratory rate spontaneous, minute ventilation total and minute ventilation spontaneous measured with the EVITA and offline flow analysis. Solid line is bias and dashed lines are 95% limits of agreement. x-axis: L/min (MVtotal and MVspontan) and respiratory rate, y-axis: percentage of difference

Data of the cluster analyses are shown in Figs. 3, 4, 5, and 6. RR_total_ is higher using the EVITA®, especially in the assisted subgroup. In the mechanical subgroup the values for RR_spont_ and MV_spont_ the EVITA® shows higher values compared to our method, especially for low MV and RR (Fig. 5). In the assisted subgroup this trend is also obvious (Fig. 6).

For lower values of RR_spont_ and MV_spont_ the difference between the two methods are larger (Figs. 5, 6).

## Discussion

In this study we developed a computerized method for respiratory flow-curve based differentiation of breathing cycle components during mechanical ventilation with superimposed spontaneous breathing. Differentiation of various phases of the breathing cycle while highlighting the relative contribution of spontaneous breathing in the overall combined respiratory rate was feasible.

Use of BIPAP/APRV enables ventilation by alternating between two positive-pressure levels, allowing unrestricted and superimposed spontaneous breathing during any phase of the respiratory cycle [21, 23, 24]. It is fundamental to understand that BIPAP/APRV can be used in different ways [25], which can result in misunderstanding and confusion about the term APRV. In this study, spontaneous breathing is unsupported unless it occurs exactly at the time at which the system changes from a lower pressure level to a higher pressure level (see Fig. 1). If spontaneous breathing occurs early in ARDS it is associated with increased ventilator free days and shorter duration of ICU [26]. Measurement of spontaneous breathing is not trivial. The exact amount of spontaneous breathing occurring during the higher pressure phase, although not supported, cannot be differentiated from the mechanical breathing portion, as the flow curve does not separate the spontaneous and the mechanical part. The only way to differentiate the two patterns would be to measure all mechanical breaths before and assume that the mechanical part will remain the same. The difference between the total tidal volume and the assumed mechanical volume would be the spontaneous breath. As the spontaneous part may have influence on the mechanical part this method is not valid in our opinion. Therefore, we labelled any spontaneous breathing during the higher pressure phase as assisted breathing. Hering and co-workers separated the breathing cycle in a short-term model into 3 phases (spontaneous assisted, spontaneous unassisted and controlled) by using a pneumotachograph and an esophageal balloon [17]. In this study, we separated the phases in a long-term animal model by using only the ventilator-measured flow curves. If validated in follow on studies in humans our method may be used at bedside and have important implications.

Assisted breaths occurred at about 45% of all breaths (Table 1), meaning that they are an entity of breaths that can’t be neglected. In addition, these assisted breaths are significantly higher in V_T_/kg as mechanical or the spontaneous breaths. If the increase in V_T_/kg of assisted breath compared to mechanical and spontaneous breath is as harmful as in pure mechanical breath remains unclear. Beside potential harmful effects on the lung, asynchrony and over breathing may influence the diaphragm. Both development of decreased, as well as of increased thickness of the diaphragm predict prolonged ventilation [13]. Exact quantification of the spontaneous breathing pattern, such as developed by us in this study, may help mitigation of the deleterious effects of over- or under breathing.

Although the use of an esophageal balloon enables monitoring of transpulmonary pressure as a surrogate for distending or driving pressure during mechanical ventilation [27, 28], we did not use the data from the esophageal balloon [18] in this study. Due to swallowing and regurgitation of the animals the position of the catheter changed often introducing potentially important inaccuracies. Using a pneumotachograph with similar measurements of flow and esophageal pressure was not possible, due to the movement of the animal and the weight of the pneumotachograph. These problems are similar to those seen in human patients, making the case for a more practical alternative which our method may well present. We used the flow signal of the ventilator to measure volume and also to differentiate the particular breath type (spontaneous, assisted or mechanical). Using this method, continuous measurement of ventilation parameters was possible without the need of additional invasive monitoring. A real-time assessment of respiration was not possible, because raw data was stored for off-line examination until after completion of the experiment. But real time measurement is absolutely mandatory for therapy of patients and estimating the assisted parts of the spontaneous breathing, to avoid potential harmful over-inflation of the lung [15, 16]. Further improvement of our algorithm may permit real time applications at the patient bedside. The driving mechanism of spontaneous breathing in ARDS is not complete clear [29] and uncontrolled extensive breathing efforts are counterproductive as they are generating high transpulmonary pressure [29]. By improving this technique and establishing an algorithm to calculate parameters directly, a bedside measurement may be possible in the future. This may give the opportunity to optimize the synchronization between the ventilator and the patient. This may be an important possible factor for improved survival in the ACURASYS study, where in severe ARDS, administration of neuromuscular blocking agents at an early time point increased ventilator free days and 90 day survival [30]. While Papazian et al. used a high sedation level and muscle paralysis to achieve the improvement in synchronization, ventilation modes with unrestricted spontaneous breathing may achieve the synchronization without potential harmful effects of sedation and muscle paralysis [31].

The only parameters that can be directly compared with the two methods in our study is RR_set_ and MV_total_ as these parameters are defined similarly. For the difference in MV_total_ there are two reasons why these are not even lower. We derived the data at the same time point as we took the data from the EVITA®. We chose to use a timespan of three minutes and usually were able to measure it 1.5 min before and after the time point. In some cases, when the data signal was not clear, we allowed a difference of 10 min to the time point. If we could not get a clear signal within 10 min the time point was not included. The data we took from the EVITA® were taken at the time of the experiment and was only one value. After getting in contact with Dräger, the timespan of the EVITA® XL to calculate their data for MV is 35 s.

In the cluster analysis we saw no differences for MV_total_ for each breathing group, while the RR_total_ seems to be higher using the EVITA®, especially in the assisted subgroup. For RR_spont_ and MV_spont_ the Bland Altmann analysis showed no clear difference in the spontaneous subgroup. In the subgroup with high parts of mechanical breaths, the values for RR_spont_ and MV_spont_ measured by the EVITA® were higher compared to our method, especially in low numbers for MV and RR. This is reasonable, as our method should have no spontaneous breathing when there is a high amount of pure mechanical breathing. In the assisted subgroup this trend is also obvious. For lower values of RR_spont_ and MV_spont_ the difference between the two methods gets larger.

The EVITA® shows in addition to MV_total_, data for MV_spont_, RR_total_, RR_spont_ and the tidal volume of each breath. The algorithm for calculation of ventilatory parameters is proprietary, and was not available for this study. In addition, these parameters are definition-dependent. With the implementation of the term assisted ventilation, including parts of spontaneous ventilation and mechanical ventilation, MV_mech_ and MV_spont_ are not directly comparable with the two methods.

### Limitations

We performed our study in an animal model so that direct transfer to human patients is not possible. The number of animals was relatively low. Differentiation of breathing phases was done manually, making subjective bias possible. To mitigate subjectivity two different persons were involved in interpretation of the flow curves.

BIPAP/APRV can be used in different ways. If the P_low_-phase is set to a pressure of zero with a short release time like described by Habashi et al. [25] the physiology of the ventilation is different compared to our study, meaning that our results cannot be transferred without caution, even if the same name of ventilation mode is used. Furthermore, our ventilation mode and ventilator setting was very specific. We did not use a pressure support or a trigger possibility, which may have led to different results. Furthermore, these data are based on our previous work comparing two groups (with and without ECCO_2_R) and thus were based on available filed from that study. Ventilation was performed similarly in both groups. Even if ECCO_2_R would have had an impact on ventilation, we think it would not affect this study, as we compared two algorithms for measurements of ventilation data.

## Conclusions

We developed a computerized method for respiratory flow-curve based differentiation of breathing cycle components during mechanical ventilation with superimposed spontaneous breathing. Further studies in humans and optimizing of this technique is necessary to allow for real-time use at the bedside.

## Electronic supplementary material

Below is the link to the electronic supplementary material.

Electronic supplementary material 1 (DOCX 455 kb)
