# Supplementary material for: Assessment of spontaneous breathing during pressure controlled ventilation with superimposed spontaneous breathing using respiratory flow signal analysis
Source: J Clin Monit Comput. 2020 Jun 13;35(4):859–68. doi: 10.1007/s10877-020-00545-4 (PMC7293172; doi:10.1007/s10877-020-00545-4)
Supplement: Supplementary file 1 — Electronic supplementary material 1 (DOCX 455 kb) [file 10877_2020_545_MOESM1_ESM.docx]

| 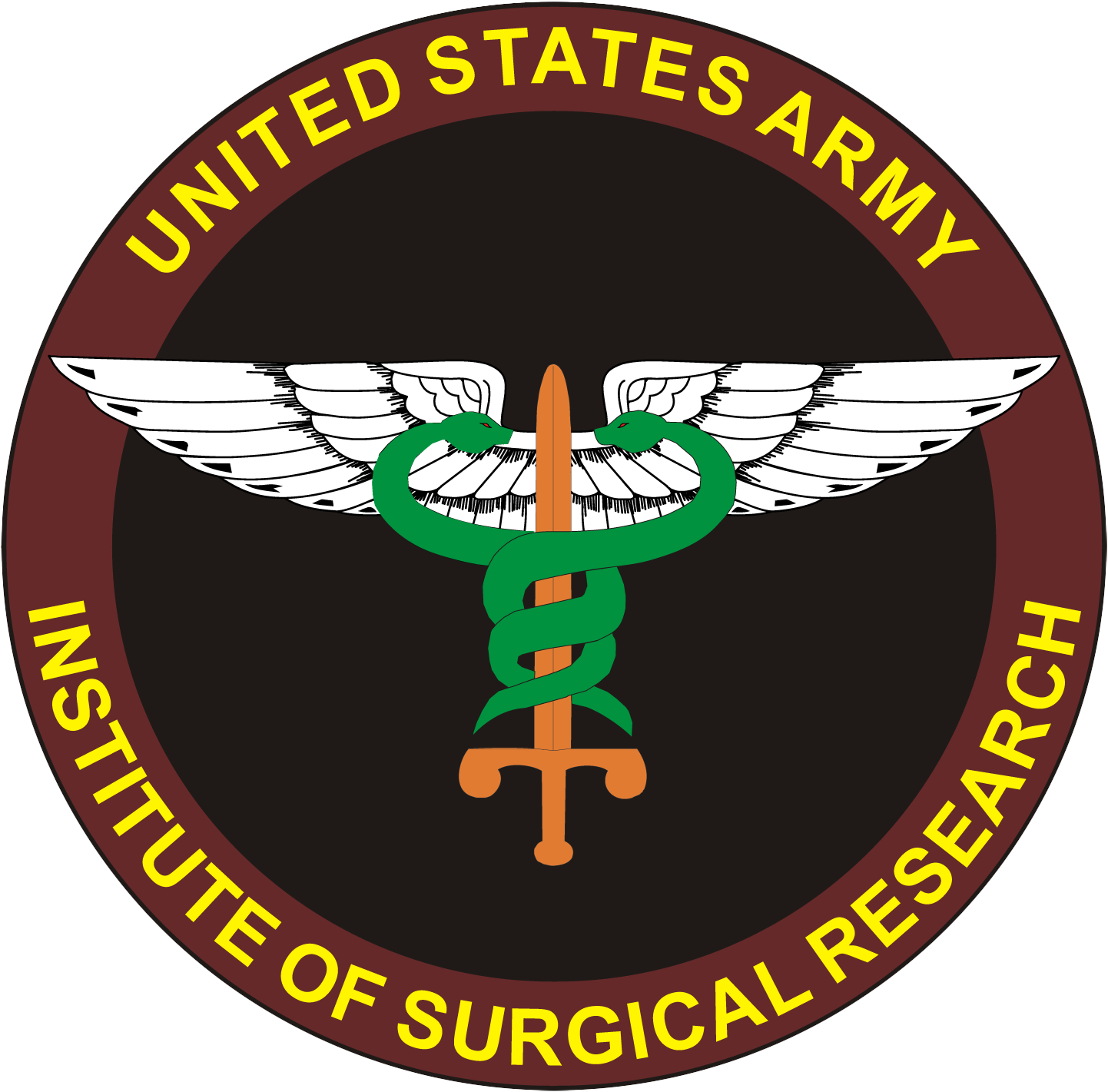 |
| --- |
| **An Algorithm for Breath Segmentation and Classification**  **Version 1.0** |
| **Medical Data Acquisition and Research Communication**  **January 2019** |
|  |

# Introduction

In this paper, we describe an algorithm (Breath-Sep) to segment a continuous stream of data from a mechanical ventilator into a series of breaths. Each breath is subdivided into three phases: inhale, exhale, and rest. The breath is further classified as spontaneous, assisted, or mechanical in nature.

The method requires two signals: the flow rate and pressure of the ventilation tube. Only the flow rate is necessary to identify the phase of the breath. The inhale phase is defined by a negative flow rate and continues until a positive flow rate is detected. A positive flow rate indicates the exhale phase of the breath. The exhale phase continues until flow decreases below a threshold value. Flow rates in the range of [-threshold,+threshold] indicate the rest phase of the breathing cycle.

To classify the breath as spontaneous, assisted, or mechanical, it is only necessary to consider the pressure during the inspiratory cycle. Three are three possibilities:

1. Pressure is low throughout the inspiratory cycle, with no pressure increase due to the ventilator. This breath, independent of the ventilator, is “Spontaneous”.
2. Pressure is nominal throughout the inspiratory cycle, which indicates the subject and ventilator are in synchronization and the breath is classified as “Assisted”.
3. Pressure increases above a nominal value and flow is primarily driven by the pressure increase of the ventilator. This indicates a “Mechanical” breath.

We tested this algorithm with data from the Drager Evita, the NM3, and the Novametrics COSMO, however, all data published was derived from the Drager Evita.

### Method

Pressure and flow rate were captured from the ventilator during treatment of a subject. Timepoints were identified on the hour for each hour of the experiment, which generally lasted two days.

For each timepoint, a 20 minute interval of data was extracted consisting of 10 minutes before the timepoint until 10 minutes after the timepoint. From the 20-minutes of extracted data, three minutes of clean data closest to the timepoint was selected and analyzed. The flow rate then analyzed to segment the breath into three states: inhale, exhale, and rest as described above.

To segment the breath into these three states, several physiological constants are assumed. First, the maximum rate of breathing was assumed to be less than 40 breaths/min, and the minimum rate of breathing was assumed to be greater than 4 breaths/min. Data segments that appeared to show breathing outside these physiological ranges was discarded. The source of the erroneous data included noise made be the animal, temporary loss of one or more signals, spurious signals, or simply unreliable signals due to noise.

For segmentation, negative flow rates indicate inhale. Inhale continues until flow rate turns positive, which marks the exhale cycle. The exhale cycle continues until flow rate decreases below a pre-determined threshold value. Flow rates below this value indicate rest before the next breath.

### Data Obtained

process_tp <- function( pfx, animal_id, tp, ivls, append_tp ) {

start_dt <- strptime(tp, '%Y%m%d_%H%M%S')

tp_file <- paste( pfx, '_', animal_id, '_', tp, sep='' )

full_name <- paste(base_dir, tp_file, sep='/')

pressure_wave <- NULL

gp2 <- NA

try ( load( full_name ), silent=TRUE )

if( is.null(pressure_wave) || is.na( pressure_wave ) ) {

cat( " --> Could not load file:", full_name, "\n" )

return( list() )

}

# cat( "load('", full_name, "')\n" )

pressure <- pressure_wave

flow <- flow_wave

if( pfx == 'C' ) {

flow <- flow + 160

pressure <- pressure + 10

}

n <- length(flow)

#

# attempt 2: calculate breath segmentation and volumes

#

sample_rate <- getSampleRate( pfx )

flow_factor <- getFlow( pfx )

xtm <- c(0:n) * (1/sample_rate) + start_dt

tt <- (cumsum( flow ) * flow_factor )

vol <- tt - cummin(tt)

rm(tt)

consec <- 0

bidx <- 0

breath_ind <- c()

breath_typ <- c()

breath_code <- c()

breath_tv <- c()

breath_tv0 <- c()

breath_tvind <- c()

is_breath = FALSE

vol0 <- vol[1]

STEPSZ <- 2

for( i in seq(2,n,STEPSZ) ) {

if( vol[ i - 1] < vol[i] ) {

consec <- consec + STEPSZ

} else {

consec <- consec - STEPSZ

}

if( consec < 0 ) {

is_breath = FALSE

consec <- 0;

}

# consecutive movements in the same direction

# and a minimum volume

if( consec > 20 && abs(vol[i-20] - vol[i]) > 10 ) {

if( !is_breath ) {

bidx <- bidx + 1

breath_ind[bidx] = i - 20

vol0 <- vol[i-20]

if( bidx > 1 ) {

pressure_seg = pressure[ breath_ind[bidx-1]:breath_ind[bidx] ]

pressure_dmax = max(pressure_seg) - pressure_seg[1]

pressure_dmin = pressure_seg[1] - min(pressure_seg)

if( pressure_dmax > 10 && pressure_dmin > 10 ) {

breath_code[bidx-1] = paste(breath_typ[bidx-1], 'a', sep='')

breath_typ[bidx-1] = 'A'

}

width = breath_ind[bidx] - breath_ind[bidx-1]

curve_len <- sum( abs(diff( pressure_seg[1:80] )) )

# cat( 'curve_len=', curve_len, ' dmax=', pressure_dmax, '\n')

if( is.na(curve_len) ) {

#hmm...ignore

} else if( curve_len > 40 ) {

breath_code[bidx-1] = paste(breath_typ[bidx-1], 'C', sep='')

breath_typ[bidx-1] = 'A'

}

if( pressure_dmax + pressure_dmin < 5 ) {

breath_code[bidx-1] = paste(breath_typ[bidx-1], 's', sep='')

breath_typ[bidx-1] = 'S'

}

}

if( i < 11 ) {

cat( "skipping eary point: ", i, "\n" )

next;

}

# flush.console()

dp = mean( pressure[ c(i-10, i+10) ] )

if( is.na( dp ) ) {

breath_typ[bidx] = '?'

} else if( dp < -3) {

breath_typ[bidx] = 'S'

} else if( dp > 10) {

breath_typ[bidx] = 'M'

} else {

breath_typ[bidx] = 'A'

# a more clear-cut assisted

}

breath_code[bidx] = ''

breath_tv[bidx] = -1

breath_tv0[bidx] = -1

breath_tvind[bidx] = -1

is_breath = TRUE

}

consec <- 20

}

if( bidx > 0 && vol[i] > breath_tv[bidx] ) {

breath_tv[bidx] <- vol[i]

breath_tv0[bidx] <- vol[i] - vol0

breath_tvind[bidx] <- i

}

}

summary <- list()

if( is.null( breath_ind) || length(breath_ind) < 3 ) {

cat('! no breath: ', tp_file, '\n')

return( summary )

} else {

breath_id <- seq( 1, length(breath_ind))

sheet_name <- paste( pfx, tp)

sheet_name <- gsub( ".rdata", "", sheet_name )

sdf <- data.frame( ID=breath_id, Tm=paste(xtm[breath_ind]), POS=breath_ind, TYP=breath_typ, TV=breath_tv0, TVx=breath_tv )

cat( ' sheet: ', xlsname, sheet_name, append_tp)

cat( '\n' )

write.xlsx2( sdf, xlsname, sheetName=sheet_name, row.names=FALSE, append=append_tp, showNA=FALSE )

}

if( with_pdf ) {

pdfname <- paste(dest_dir, '/pdf/', pfx, '-', animal_id, '-', tp, '.pdf', sep='' )

pdfname <- gsub( ".rdata", "", pdfname )

pdf( file=pdfname, width=8.5, height=11 )

}

par( mfrow=c(9,1) )

par( mar=c(2,4,1,1) )

for( m in ivls ) {

mv0 <- show_min( pfx, xtm, m, m + 0.5, pressure, flow, vol, breath_ind, breath_tv0, breath_tvind, breath_typ, breath_code, gp2_wave )

mv1 <- show_min( pfx, xtm, m + 0.5, m + 1, pressure, flow, vol, breath_ind, breath_tv0, breath_tvind, breath_typ, breath_code, gp2_wave )

plot.new()

text(0.1, 0.8, paste('Animal:', animal_id ), adj=0, font=2 )

text(0.1, 0.6, paste('Time:', start_dt + 60*m ), adj=0, font=2 )

text(0.1, 0.4, paste('Source:', pfx ), adj=0, font=2 )

text(0.7, 1, 'Expiratory', adj=0 )

text(0.85, 1, 'Inspiratory', adj=0 )

text(0.7, 0.8, 'Mech MV: ', adj=1 )

text(0.7, 0.6,'Spont MV: ', adj=1 )

text(0.7, 0.4,'Assisted MV: ', adj=1 )

text(0.7, 0.1,'Total MV: ', adj=1, font=2 )

text(0.72, 0.8, format(mv0$mech_e + mv1$mech_e, digits=3), adj=0 )

text(0.85, 0.8, format(mv0$mech_i + mv1$mech_i, digits=3), adj=0 )

text(0.72, 0.6, format(mv0$spont_e + mv1$spont_e, digits=3), adj=0 )

text(0.85, 0.6, format(mv0$spont_i + mv1$spont_i, digits=3), adj=0 )

text(0.72, 0.4, format(mv0$assist_e + mv1$assist_e, digits=3), adj=0 )

text(0.85, 0.4, format(mv0$assist_i + mv1$assist_i, digits=3), adj=0 )

text(0.72, 0.1, format(mv0$tot_e + mv1$tot_e, digits=3), adj=0, font=2 )

text(0.85, 0.1, format(mv0$tot_i + mv1$tot_i, digits=3), adj=0, font=2 )

summary <- rbind( summary, mv0, mv1 )

}

if( with_pdf ) {

dev.off()

}

return( summary )

}
